# Bericht der Arbeitsgemeinschaft Kinder- und Fetalpathologie der Deutschen Gesellschaft für Pathologie

**DOI:** 10.1007/s00292-024-01358-x

**Published:** 2024-09-26

**Authors:** Elise Gradhand, Thomas Hager

**Affiliations:** 1 Dr. Senckenbergisches Institut für Pathologie, Sektion Kinder und Perinatalpathologie, Universitätsklinikum Frankfurt/Main, Frankfurt, Deutschland; 2 Institut für Pathologie, MVZ DIAKO Flensburg, Flensburg, Deutschland

Am Samstag, den 25. Mai 2024, fand die beiden Sitzungen der AG Kinder- und Fetalpathologie in München im Rahmen der 107. Jahrestagung der Deutschen Gesellschaft für Pathologie statt. Es wurden verschiedene aktuelle Forschungsergebnisse und klinische Studien aus dem Gebiet der Kinder und Perinatalpathologie präsentiert.

## 1. Sitzung

Zu Beginn der ersten Sitzung präsentierten die eingeladenen Referenten R. Hiller und L.-C. Horn (Leipzig) im Hauptvortrag diagnostische Ansätze zur Molenschwangerschaft und deren Differentialdiagnosen. Sie gingen auf die Bedeutung der Histopathologie bei der Unterscheidung zwischen verschiedenen trophoblastären Läsionen und retentionsbedingten Veränderungen ein. Diese Unterscheidung ist entscheidend für die Therapie und klinische Diagnostik bei gestationsbedingten Trophoblasterkrankungen. Anschließend stellte die Humangenetikerin C. Hendrich (Hannover) ihre wegweisende Studie „Post-mortem trio genome sequencing in families with children or adolescents with an unexplained cause of death“ vor. Die Untersuchung fokussierte sich auf die Möglichkeiten der genetischen Diagnostik nach dem Tod von Kindern mit unklaren Todesursachen. Erste Ergebnisse deuten darauf hin, dass postmortale genetische Analysen wertvolle Informationen liefern können, um Verdachtsdiagnosen zu bestätigen und Versorgungslücken aufzudecken.

## 2. Sitzung

In der zweiten Sitzung präsentierten N. Schaumann und J.-T. Suhren (Hannover) ihre diagnostisch sehr relevante Arbeit „Placenta pathology in COVID-19-pregnancies“, in der festgestellt wurde, dass spezifische Plazentapathologien bei COVID-19-assoziierten Schwangerschaften nicht häufiger auftreten als bei Nicht-COVID-19-Fällen. Ein weiterer innovativer Beitrag kam von S. Gretser (Frankfurt a.M.) mit einer Studie, die die Rolle der Fluoreszenz-Konfokalmikroskopie bei der Bewertung der Vitalität von pädiatrischen Tumorproben untersucht. Es zeigte sich, dass diese Methode geeignet ist, um vitale Tumorareale in Echtzeit zu identifizieren, ohne die Kultivierung von Tumorzellen zu beeinträchtigen. Im weiteren Verlauf der Sitzung beleuchteten T. Manuylova (Tübingen) in ihrem Beitrag „Congenital gastrointestinal malformations: Pathological perspectives and diagnostics“ die Bedeutung der autoptischen Untersuchung bei der klinischen Diagnose von gastrointestinalen Fehlbildungen bei Neugeborenen. A. M. Müller (Köln) stellte ihre Forschung zu „Single-Shot Cardioplegia: Del Nido or HTK solution?“ vor, in der die Effizienz zweier kardioprotektiver Lösungen bei Neugeborenen verglichen wurde. Die Ergebnisse zeigten, dass die HTK-Lösung eine bessere strukturelle Schutzwirkung für das Neugeborenenherz bietet als die Del-Nido-Lösung, insbesondere bei längerer Ischämiezeit. J.-T. Suhren (Hannover) zeigte in seinem Vortrag den Vergleich von fetaler Autopsie und pränataler Diagnostik. Es wurde festgestellt, dass pränatale Ultraschallbefunde in den meisten Fällen mit den Autopsieergebnissen übereinstimmen. Abschließend stellte M. Overkamp (Tübingen) in einem Vortrag „Viral Myocarditis in Childhood“ die Bedeutung der histopathologischen und molekularen Diagnostik bei kindlicher Myokarditis hervor, da nach wie vor häufig die exakte histologische Diagnose entscheidend für die Wahl der Therapie ist und somit das klinische Ergebnis erheblich beeinflusst.

In beiden Sitzungen fanden sehr interessante und konstruktive Diskussionen zwischen den Referenten und dem Auditorium statt, die das Verständnis der vorgestellten Themen aus dem Bereich der Kinder- und Perinatalpathologie vertieften und zu neuen Einsichten in aktuelle Entwicklungen gaben.

### Mitgliederversammlung

Bei der sich anschließenden Mitgliederversammlung wurden mehrere aktuelle Themen besprochen und beschlossen, diese in einer ausführlichen Mitgliederversammlung zur Gemeinsamen Herbsttagung der AG Kinderpathologie und der Fachgesellschaft der Kinderpathologie am 27./28. September 2024 in Tübingen weiter zu diskutieren.

